# Validation of a Simple, Rapid, and Cost-Effective Method for Acute Rejection Monitoring in Lung Transplant Recipients

**DOI:** 10.3389/ti.2022.10546

**Published:** 2022-06-09

**Authors:** Monica Sorbini, Gabriele Togliatto, Fiorenza Mioli, Erika Simonato, Matteo Marro, Margherita Cappuccio, Francesca Arruga, Cristiana Caorsi, Morteza Mansouri, Paola Magistroni, Alessandro Gambella, Luisa Delsedime, Mauro Giulio Papotti, Paolo Solidoro, Carlo Albera, Massimo Boffini, Mauro Rinaldi, Antonio Amoroso, Tiziana Vaisitti, Silvia Deaglio

**Affiliations:** ^1^ Department of Medical Sciences, University of Turin, Turin, Italy; ^2^ Cardiac Surgery Division, Surgical Sciences Department, Heart and Lung Transplant Center, Città Della Salute e Della Scienza University Hospital, Turin, Italy; ^3^ Immunogenetics and Transplant Biology Service, Città Della Salute e Della Scienza University Hospital, Turin, Italy; ^4^ Pathology Unit, Città Della Salute e Della Scienza University Hospital, Turin, Italy; ^5^ Department of Oncology, University of Turin, Turin, Italy; ^6^ Lung Transplantation and Advanced Airways Management, Città Della Salute e Della Scienza University Hospital, Turin, Italy

**Keywords:** lung transplantation, biomarker, acute rejection, cell free circulating DNA, droplet-digital PCR

## Abstract

Despite advances in immunosuppression therapy, acute rejection remains the leading cause of graft dysfunction in lung transplant recipients. Donor-derived cell-free DNA is increasingly being considered as a valuable biomarker of acute rejection in several solid organ transplants. We present a technically improved molecular method based on digital PCR that targets the mismatch between the recipient and donor at the *HLA-DRB1* locus. Blood samples collected sequentially post-transplantation from a cohort of lung recipients were used to obtain proof-of-principle for the validity of the assay, correlating results with transbronchial biopsies and lung capacity tests. The results revealed an increase in dd-cfDNA during the first 2 weeks after transplantation related to ischemia-reperfusion injury (6.36 ± 5.36%, *p* < 0.0001). In the absence of complications, donor DNA levels stabilized, while increasing again during acute rejection episodes (7.81 ± 12.7%, *p* < 0.0001). Respiratory tract infections were also involved in the release of dd-cfDNA (9.14 ± 15.59%, *p* = 0.0004), with a positive correlation with C-reactive protein levels. Overall, the dd-cfDNA percentages were inversely correlated with the lung function values measured by spirometry. These results confirm the value of dd-cfDNA determination during post-transplant follow-up to monitor acute rejection in lung recipients, achieved using a rapid and inexpensive approach based on the HLA mismatch between donor and recipient.

## GRAPHICAL ABSTRACT

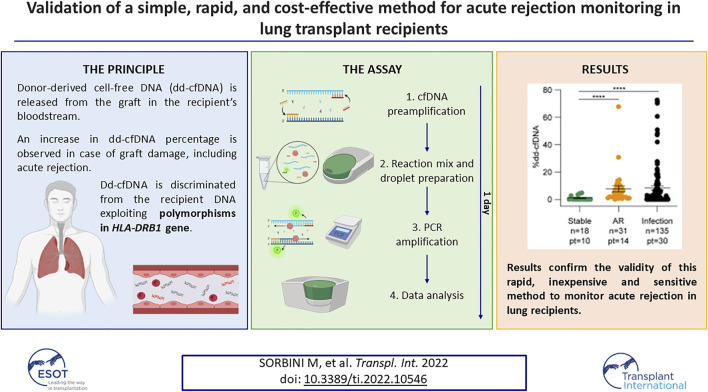

## Introduction

Affecting almost one patient in three between discharge and 1-year follow up, acute rejection (AR) represents one of the most common causes of allograft dysfunction after lung transplantation (1). If not promptly recognized and treated, AR can lead to chronic lung allograft dysfunction (CLAD), significantly reducing patient survival (2-4). In contrast, inappropriate treatment of AR episodes with immunosuppressive drugs to limit organ damage can significantly increase the risk of infections, which can be a potentially lethal complication in lung transplant recipients (5, 6). Overall, while advances in immunosuppression regimens have improved 1-year survival to >90%, 5-year survival remains around 50% (7, 8).

Bronchoscopy with associated transbronchial lung biopsy (TBB) and cytology are typically used to monitor acute cellular rejection (ACR), whereas analysis of donor-specific antibodies (DSA) in recipients’ sera detects antibody-mediated rejection (AMR). However, even if both techniques are currently the “gold standard” in rejection monitoring, they can be poorly informative. First, TBB is invasive with possible complications, whereas DSA only detects anti-HLA antibodies, limiting their clinical impact and stressing the need for additional tools in post-transplant monitoring (7, 9-11).

Donor-derived cell-free DNA (dd-cfDNA) has recently been proposed as a biomarker for graft injury (12). DNA is released from donor cells because of allograft damage and can be detected in the recipient bloodstream. Dd-cfDNA levels increase during acute rejection episodes according to the severity of damage in many solid organ transplants (12-16). In lung transplant recipients, donor DNA levels were found to increase during acute rejection episodes (5, 17, 18) and during respiratory tract infections (5, 19, 20).

Donor DNA can be distinguished from the recipient DNA by using single nucleotide polymorphisms (SNPs). The most sensitive techniques are based on the simultaneous evaluation of dozens of SNPs using next-generation sequencing (NGS), guaranteeing high accuracy (13, 14, 21). However, NGS-based techniques are very expensive (13, 21, 22) and usually need to be analyzed in pools, implying that a single sample may be waitlisted until a set number is reached, representing significant limitations to its widespread application.

We have previously optimized a simple method to quantify dd-cfDNA based on genetic differences between the donor and host at the *HLA-DRB1* locus, which is routinely analyzed before transplantation. The assay, which is based on a droplet digital PCR technique, is more rapid, technically easier, and significantly less expensive than NGS-based analysis of dozens of SNPs. The results obtained in a cohort of heart-transplanted patients show that this assay is effective in identifying patients undergoing rejection, with 64.2% sensitivity and 70.8% specificity (23). Here, we present the results of a dd-cfDNA analysis from a cohort of lung transplant recipients performed with an improved version of the test. This now exploits two panels of probes targeting *HLA-DRB1*, labelled with two different fluorophores, with increased sensitivity and lower costs.

## Materials and Methods

### Patient Recruitment

This study was approved by the Ethics Committee of Città della Salute e della Scienza University Hospital of Turin (approval #CS2/1202, 9 April 2019). The enrolled patients underwent lung transplantation from 1 July 2019, to 31 March 2021, and provided written informed consent. Exclusion criteria were refusal or inability to provide informed consent, any form of substance abuse, psychiatric disorders, or conditions that could complicate communication between the investigator and the patient. Patient data were anonymized using an alphanumeric ID and all sensitive information was conserved on the RedCap online platform (https://www.medcap.unito.it/redcap/index.php) and used for analysis.

### Donor and Recipient HLA Typing

Donor and recipient HLA typing was performed by the Immunogenetics and Transplant Biology Service, Città della Salute e della Scienza University Hospital of Turin, as routine management. Patients were HLA-typed by Luminex using the LabType SSO and LabType SSO XR kits (One Lambda, Inc., West Hills, CA, United States). Donor HLA loci were assessed by real-time PCR using a LinkSeq HLA typing kit (One Lambda, Inc., West Hills, CA, United States). Donor and recipient pairs sharing the same *HLA-DRB1* allele were excluded from the analysis.

### Transplantation

According to a national protocol, urgent lung transplantation was reserved for young patients (age ≤ 50 years) requiring mechanical ventilation and/or extracorporeal lung support with extracorporeal membrane oxygenation (ECMO) (24). The graft was preserved with an anterograde and retrograde flush using Perfadex and stored at 4°C. Grafts considered unsuitable for direct transplantation underwent *ex vivo* lung perfusion (EVLP) prior to transplantation, performed according to the Toronto technique (25). Lungs from two donors recovered after cardiac death (DCD) in the Maastricht III group (26). Lung transplantation was performed according to standard techniques. Cardiopulmonary bypass was used in cases of poor oxygenation on monolateral ventilation, hemodynamic instability after pulmonary artery clamping, or in patients on extracorporeal ventilation before transplant.

### Post-Transplant Clinical Management

All patients were admitted to a dedicated intensive care unit ICU, allowing controlled ventilator weaning. Primary graft dysfunction (PGD), defined according to the International Society of Heart and Lung Transplantation (ISHLT) guidelines (27), was evaluated at the time of admission to the ICU and after 24 and 72 h. Immunosuppressive therapy during the induction phase included thymoglobulin (1 mg/kg/day for 5 days) and steroids. Immunosuppression maintenance was based on calcineurin inhibitors (mainly cyclosporine), antimetabolites (mycophenolate), and corticosteroids. After discharge, patients were followed up in our lung transplant day hospital using spirometry, blood gas analysis, and medical and radiologic examinations to assess lung function. Rejection events determined by histological and clinical examination were mainly treated with pulse dose glucocorticoids (methylprednisolone 15/mg/kg/day for 3 days). CLAD was defined as a substantial and persistent decline (≥20%) in the forced expiratory volume in 1 s (FEV1) when compared with the post-transplant baseline (28), and based on its duration classified as possible (<3 weeks), probable (≤3 months) and definite (>3 months). Biochemical and microbiological evaluations on blood and bronchoalveolar lavage samples were performed routinely and in case of suspicion of bacterial and viral infections.

### Sample Collection

Plasma samples were collected using PAXgene Blood ccfDNA tubes (#768165; Qiagen, Hilden, Germany). Blood samples were collected weekly during hospitalization following transplantation, and then every time the patients underwent medical examinations or transbronchial biopsies. A total of 372 plasma samples were obtained from 30 patients (average, 12,4/patient). Plasma was separated by centrifugation (2,000 ×g, 15 min, 18°C) and stored at −80°C in the Teseo Biobank of the Department of Medical Sciences of University of Turin (http://www.progettoeccellenzateseo.unito.it/) until further processing. Cell-free DNA (cfDNA) was extracted from 1 ml of plasma using MagMAX Cell free DNA isolation kit (#A29319, Applied Biosystems, Waltham, MA, United States) and stored at −20°C until analysis**.**

### Donor DNA Quantification

Two nanograms of cfDNA was amplified using Sso PreAmp Assays (#1725160, Bio-Rad, Hercules, CA, United States) to enrich the number of *HLA-DRB1* gene molecules, and 2 µl of the amplified product was used in the following step. Dd-cfDNA was quantified using an Expert Design assay probe panel (Bio-Rad, Hercules, CA, United States) designed to target the *HLA-DRB1* gene. A list of the available probes is reported in Supplementary Table S1. Donor and recipient DNA were amplified using specific primers and probes labelled with FAM and HEX fluorescent dyes, respectively. Droplet digital PCR reaction mix included 11 µl of ×2 droplet digital PCR Supermix for Probes no UTP (#186-3023, Bio-Rad, Hercules, CA, United States), 1.1 µl of Bio-Rad Expert Design assay FAM probe and 1.1 µl of Bio-Rad Expert Design assay HEX probe (final volume 20 µl). Droplet generation and amplification were performed as reported before (19, 23). Donor DNA was quantified as the ratio between donor and total copies and was expressed as a percentage. All measurements were performed in triplicates.

### Histopathology

Surveillance lung allograft bronchoscopy and TBBs were performed at 4, 8, 12- and 18-month post-transplant. In addition, bronchoscopy and TBB were performed whenever there was clinical suspicion of rejection or pulmonary infection. The Working Formulation of the ISHLT criteria (29) was applied by experienced transplant pathologists to diagnose and grade all graft TBBs. In particular, the diagnosis of AR is based on the presence of perivascular and interstitial inflammatory cell infiltrates. Subendothelial infiltration/endotheliitis was also considered relevant for the final diagnosis of AR. Based on the histological extent of injury and inflammation, AR was graded as absent (grade A0), minimal (grade A1), mild (grade A2), moderate (grade A3), or severe (grade A4). Grade A2 is generally considered a threshold for therapeutic intervention. Morphological (e.g., neutrophilic margination, neutrophilic capillaritis, and acute lung injury with or without hyaline membrane deposits) and immunohistochemical (i.e., C4d deposition in interstitial alveolar capillaries) features of AMR were assessed and graded according to ISHLT and Banff recommendation (30-32).

### DSA Evaluation

Sixty serum samples were collected at the time of liquid biopsy during posttransplant management. Sera were assessed for DSA by Luminex using commercially available SAB kits (LSM12, LS2A01, and LSA104 assays, One Lambda, West Hills, CA, United States), and the results were expressed as mean fluorescence intensity (MFI, cut-off positive value > 1,000). All the patients were DSA-negative at the time of transplantation. In addition, all patients had a negative cross-match, as determined by flow cytometry (FACSLyric, BD Biosciences) testing of sera for the presence of IgG and IgM antibodies against donor T and B lymphocytes isolated from peripheral blood samples.

### Statistical Analysis

Dd-cfDNA quantification is reported as mean ± standard deviation (SD). Differences between mean values in each group were compared using the Mann-Whitney nonparametric test, since data resulted to be not normally distributed using the Shapiro Wilk test. *p*-values lower than 0.05 were considered as statistically significant. The correlation between two continuous variables was analyzed using the nonparametric Spearman test. Receiver operating characteristic (ROC) curves were calculated using the Wilson-Braun method. All statistical analyses were performed using GraphPad Prism version8.0.2.

## Results

### Validation of Expert Design Assay Probe Panel

**FIGURE 1:**
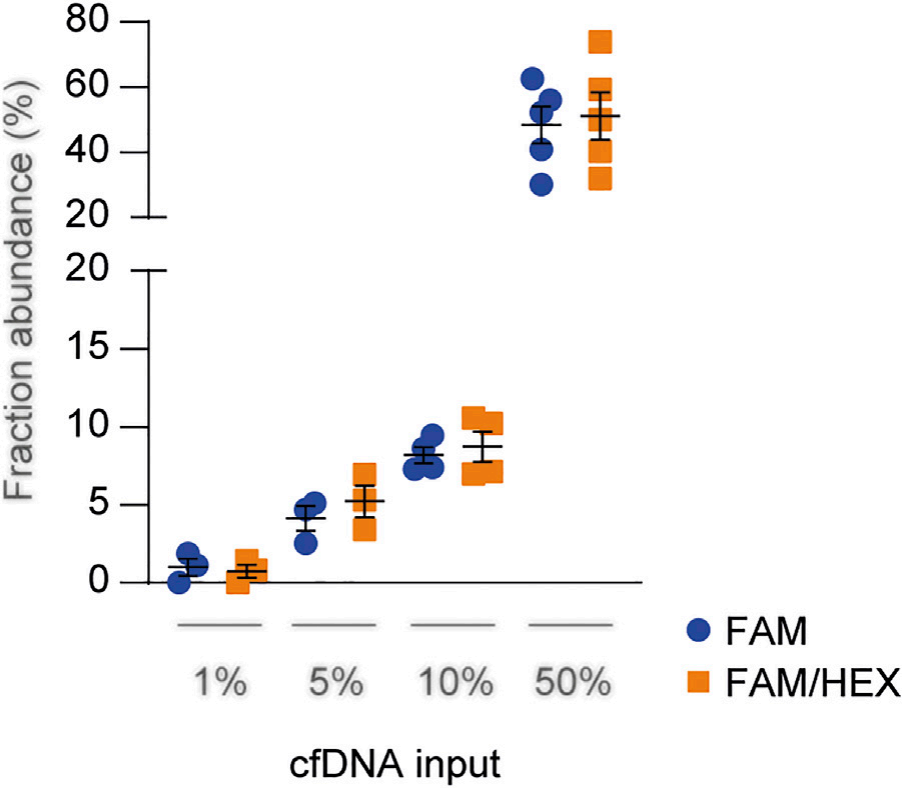
Technical comparison between *HLA-DRB1* FAM and FAM/HEX probe panels. Serially diluted cfDNAs were spiked into a constant level of background cfDNA and quantified through droplet digital PCR assay using both the FAM-only and FAM/HEX methods. The total DNA concentration was 10 ng and the percentage of spiked DNA is shown in the graph. The results were reported as the mean fraction abundance. Error bars represent SEM. *p*-values were obtained using the Mann-Whitney nonparametric test.

The sensitivity and specificity of *HLA-DRB1* FAM probes have been assessed previously assessed (19, 23). The HEX panel was validated by testing each probe with several cfDNAs to assess specificity, and by performing serial dilution to determine sensitivity (Supplementary Figure S1). Combinations of cfDNA (Figure 1) or genomic DNA (data not shown) carrying different *HLA-DRB1* alleles were loaded at known concentrations (1%, 5%, 10%, and 50%) and quantified using the FAM-only probe method described before (23), or by combining FAM and HEX probes targeting the two alleles in the same reaction. Since the results were consistent between both methods, we concluded that dd-cfDNA quantification of both the donor and recipient in the same well was feasible, with a reduction in time and costs of analyses while maintaining comparable accuracy.

### Patients’ Characteristics

Thirty consecutive patients who underwent primary lung transplantation at our institution between 1st July 2019 and 31st March 2021 were recruited for this study (Table 1). In 28 out of 30 cases (93.3%), organs were recovered from heart-beating donors (13 males, 43.3%) with a mean age at death of 42.5 ± 16.1. In the two remaining cases, donations occurred after circulatory death in patients aged 58 and 69 years, respectively. The mean waiting list time was 306.0 (range: 3–1,607) days, with a median of 226.5 days. The mean age at transplantation was 47.0 ± 15.5. Twenty-one patients (70.0%) received a double lung transplant, four (13.3%) received a single lung transplant, and five (16.7%) received a bilateral lung transplant associated with another solid organ transplantation (one lung-heart, one lung-kidney, and three lung-liver-pancreas). Idiopathic pulmonary fibrosis (11 cases, 36.7%) was the most common disease, followed by cystic fibrosis (7 patients, 23.3%), and chronic obstructive pulmonary disease (6 cases, 20.0%). Nine patients (30.0%) received a transplant on an urgent basis, four (13.3%) received mechanical ventilation, and 6 (20.0%) received ECMO before transplantation. 22 subjects (73.3%) presented with clinical signs of primary graft dysfunction (PGD) of any grade within the first 72 h after transplantation. Three patients (10.0%) experienced grade 3 PGD 72 h after transplant. Lastly, four recipients (13.3%) received organs that underwent EVLP before transplantation. The mean total organ ischemia was 332.9 ± 159.4 min. The median total hospital stay was 47.5 days, and none of the patients died before discharge.

**TABLE 1:** Donors and recipients’ characteristics. List of main features of donors and recipients included in the study. The number and percentage of subjects in each group are shown.

	Variable	Monopulmonary LTx	Bipulmonary LTx	Combined LTx	Total
		*N* = 4	*N* = 21	*N* = 5	*N* = 30
Donor	Age (y), mean ± SD	41.1 ± 17.6	42.1 ± 16.2	41.8 ± 16.8	42.5 ± 16.1
	Male sex, n (%)	3 (75.0)	7 (33.3)	3 (60.0)	13 (43.3)
	Cardiac death, n (%)	0 (0)	2 (9.52)	0 (0)	2 (6.70)
	Brain death, n (%)	4 (100)	19 (90.5)	5 (100)	28 (93.3)
	Ischemic time (minutes), mean ± SD	352.0 ± 181.1	331.0 ± 157.5	342.4 ± 170.8	332.9 ± 159.4
Recipient	Age (y), mean ± SD	48.2 ± 17.2	47.0 ± 15.7	46.7 ± 17.1	47.0 ± 15.5
	Male sex, *n* (%)	4 (100)	7 (33.3)	4 (80.0)	15 (50.0)
	Disease, *n* (%)				
	IPF	3 (75.0)	8 (38.1)	0 (0)	11 (36.7)
	CF	0 (0)	4 (19.0)	3 (60.0)	7 (23.3)
	COPD	0 (0)	5 (23.8)	1 (20.0)	6 (20.0)
	BOS	1 (25.0)	1 (4.8)	0 (0)	2 (6.7)
	Ciliary dyskinesia	0 (0)	2 (9.5)	0 (0)	2 (6.7)
	PH	0 (0)	1 (4.8)	1 (20.0)	2 (6.7)
	Total hospital stay (d), mean ± SD	67.8 ± 59.0	67.8 ± 54.8	68.3 ± 57.8	66.7 ± 54.2
	CEC, *n* (%)	0 (0)	9 (42.9)	2 (40.0)	11 (36.7)
	ECMO, *n* (%)	0 (0)	10 (47.6)	2 (40.0)	12 (40.0)
	EVLP, *n* (%)	0 (0)	4 (19.0)	0 (0)	4 (13.3)
	Hemodynamic support, *n* (%)	3 (75.0)	19 (90.5)	5 (100)	27 (90.0)
	Dobutamine	0 (0)	11 (52.4)	5 (100)	13 (43.3)
	Noradrenaline	3 (75.0)	15 (71.4)	1 (20.0)	19 (63.3)
	iNO	1 (25.0)	5 (23.8)	2 (40.0)	8 (26.7)
	Pulmonary infections, *n* (%)	4 (100)	21 (100)	5 (100)	30 (100)
	Bacteria	1 (25.0)	18 (85.7)	5 (100)	24 (80.0)
	Virus	3 (75.0)	18 (85.7)	2 (40.0)	23 (76.7)
	Fungi	1 (25.0)	8 (38.1)	2 (40.0)	11 (36.7)

LTx, lung transplant; SD, standard deviation; IPF, idiopathic pulmonary fibrosis; CF, cystic fibrosis; COPD, chronic obstructive pulmonary disease; BOS, bronchiolitis obliterans; PH, pulmonary hypertension; CEC, extracorporeal circulation; ECMO, extracorporeal membrane oxygenation; EVLP, *ex vivo* lung perfusion; iNO, inhaled nitric oxide.

### Donor-Derived Cell-Free DNA Release Is Influenced by Ischemia-Reperfusion Injury

**FIGURE 2:**
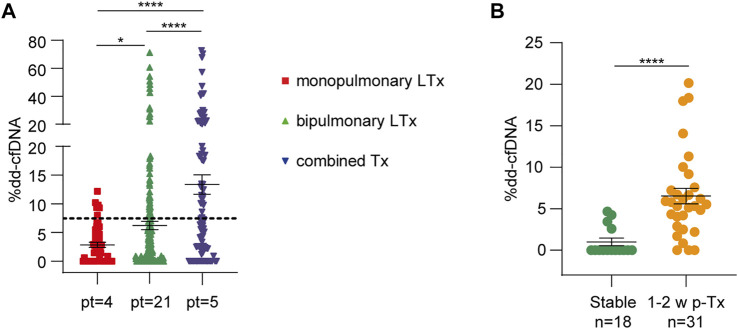
Dd-cfDNA release is influenced by the type of lung transplant and ischemia-reperfusion injury. **(A)** Dd-cfDNA quantification in monopulmonary, bipulmonary, and combined lung transplants (LTx). The number of patients (pt) is reported for each group. The dotted line represents the total average percentage of dd-cfDNA. **(B)** dd-cfDNA levels during the first 2 weeks after transplantation (31 measurements) were compared to stable condition samples (18 measurements from 10 patients). The number of samples (n) in each group is shown below. The results are reported as percentages and shown as dot plots. Error bars represent SEM. *p*-values were obtained using the Mann-Whitney nonparametric test.

In total, 372 plasma samples were obtained from 30 patients (mean 12, 4 samples/patient). The mean dd-cfDNA percentages obtained at all times differed significantly between monopulmonary, bipulmonary, and combined transplant recipients, reflecting the number of donor cells present in the recipient (Figure 2A). In fact, mean donor DNA levels were lower in single-lung recipients (2.8 ± 3.2%) than in double-lung (6.2% ± 10.9%, *p* = 0.02) or combined transplant recipients (13.3% ± 16.2%, *p* < 0.0001). During the first 2 weeks after transplantation, dd-cfDNA peaked (mean value 6.36 ± 5.36%), in line with previous results, demonstrating organ damage due to ischemia-reperfusion (Figure 2B). In patients without complications, the mean donor dd-cfDNA quantification slowly stabilized at 2 weeks after transplantation. To determine the baseline value to be used for comparisons, we selected 18 samples from 10 patients (one monopulmonary, one combined, and eight bipulmonary recipients) at a time when no sign of rejection, infection, or worsening of their clinical condition could be observed. The mean dd-cfDNA calculated from these samples (2.18% ± 3.26%) was considered as the baseline.

### Acute Rejection Is Followed by a Significant Increase of Donor-Derived Cell-Free DNA

**FIGURE 3:**
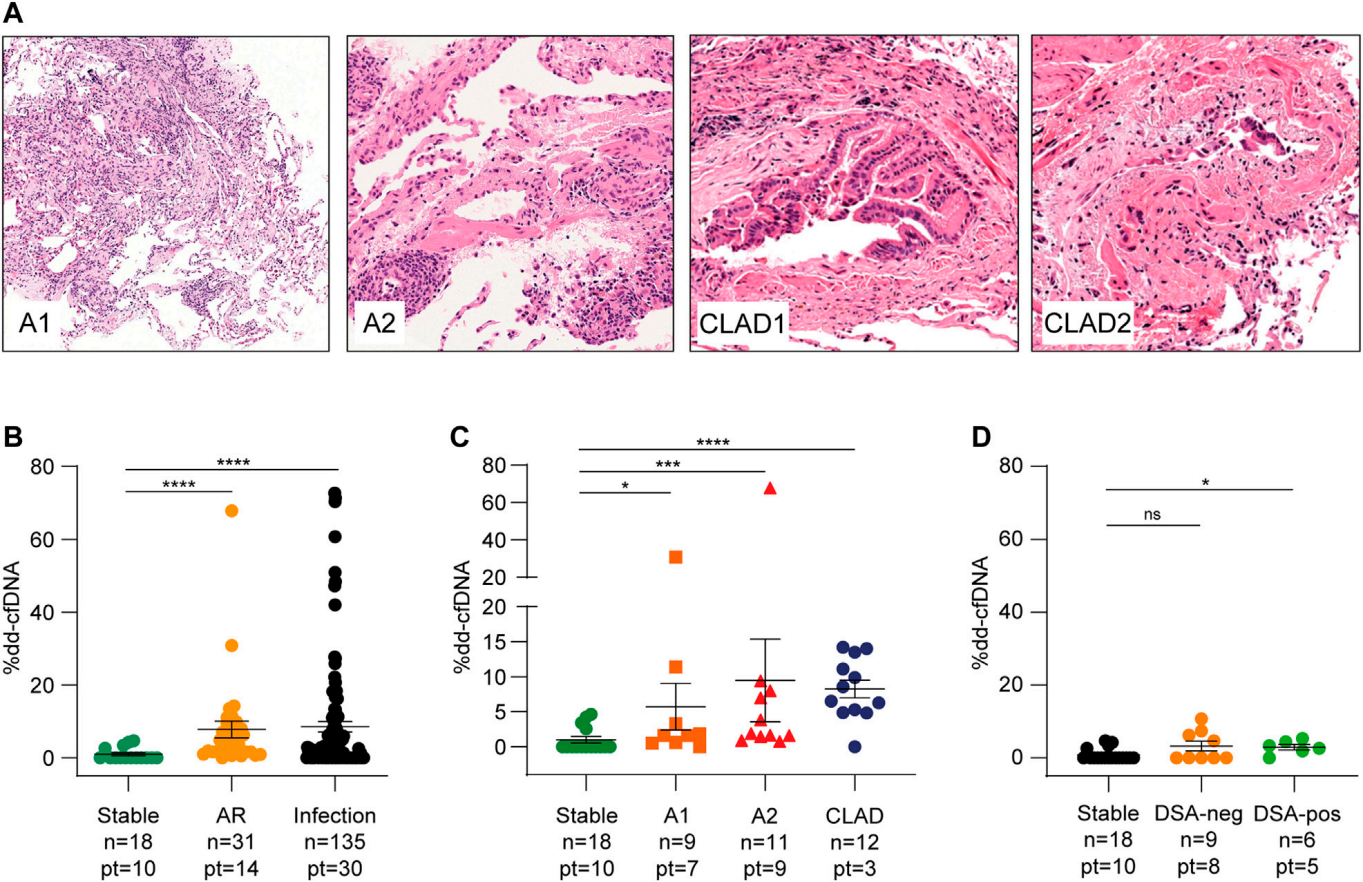
Acute rejection is followed by a significant increase of dd-cfDNA. **(A)** Histopathological features of acute rejection grades A1 and A2 and evidence of bronchiolar wall fibrosis with lumen narrowing (CLAD1) and epithelial damage (CLAD2) in patients with obliterative bronchiolitis syndrome (BOS-CLAD). Hematoxylin and eosin staining, A1 ×100 original magnification, A2 and CLAD ×200 original magnification. **(B)** dd-cfDNA values during acute rejection (AR) and infectious events compared to stable conditions. **(C)** donor DNA levels in minimal (A1) and mild (A2) rejection and in chronic lung allograft dysfunction (CLAD) episodes. **(D)** dd-cfDNA percentages in DSA-negative and DSA-positive samples compared to those under stable conditions. The numbers of samples (n) and patients (pt) in each group are indicated. The results are reported as percentages and shown as dot plots. Error bars represent SEM. *p*-values were calculated using the Mann-Whitney non-parametric test.

A total of 20 out of 115 transbronchial biopsies (17.4%) scored positive for cellular rejection. Nine biopsies were classified as minimal grade (indicated as A1) and 11 as mild grade (indicated as A2, Figure 3A). No grade ≥ A3 biopsies were observed during the follow-up period. Donor DNA levels were more elevated during AR events than under stable conditions (7.81 ± 12.7%, *p* < 0.0001, Figure 3B and Supplementary Figure S2). In addition, levels varied significantly according to the severity of rejection; A1 events were related to a modest increase in donor DNA amount (mean value 5.74 ± 10.0%, *p* = 0.03), whereas A2 rejection caused a stronger increase (9.48 ± 19.60%, *p* = 0.008, Figure 3C). No biopsy showed morphological or immunohistochemical features of AMR (Supplementary Figure S3), even though five patients (16.7%) developed DSA after transplantation and two of them had anti-HLA-DQ antibodies, which are generally associated with AMR. Of the 60 DSA tests performed, 38 (63.3%) were negative in accordance with negative biopsies, 2 (3.3%) were positive and associated with biopsy-proven A2 rejection, and 15 (25.0%) did not agree with the histochemical evaluation. The remaining five (8.4%) samples were not temporally related to graft tissue collection. Donor DNA percentages obtained from seven samples temporally close to DSA-positive sera were higher than those obtained from DSA-negative samples (nine samples). To avoid confounding factors that could affect the analysis, this statistical evaluation was performed considering only serum samples collected in the absence of documented infections and other evidence of graft damage not due to rejection (16 samples). Even if the number of samples included in the statistical analysis was limited, results reached significance when comparing stable conditions vs. DSA-positive (*p* = 0.01, Figure 3D). On the contrary, we could not observe significant differences between DSA-positive and DSA-negative samples. Finally, three patients (10.0%) experienced possible CLAD, two of whom showed clinical signs of bronchiolitis obliterans (BOS) and then recovered (Figure 3A), whereas the remaining patient developed a mixed form of BOS and restrictive allograft syndrome and died from severe pulmonary insufficiency caused by chronic rejection and pneumonia. All samples collected during these episodes showed elevated levels of dd-cfDNA (8.26 ± 4.41%, *p* < 0.0001, Figure 3C).

### Respiratory Tract Infections Were Related to Significant Changes in Donor-Derived Cell-Free DNA Levels

**FIGURE 4:**
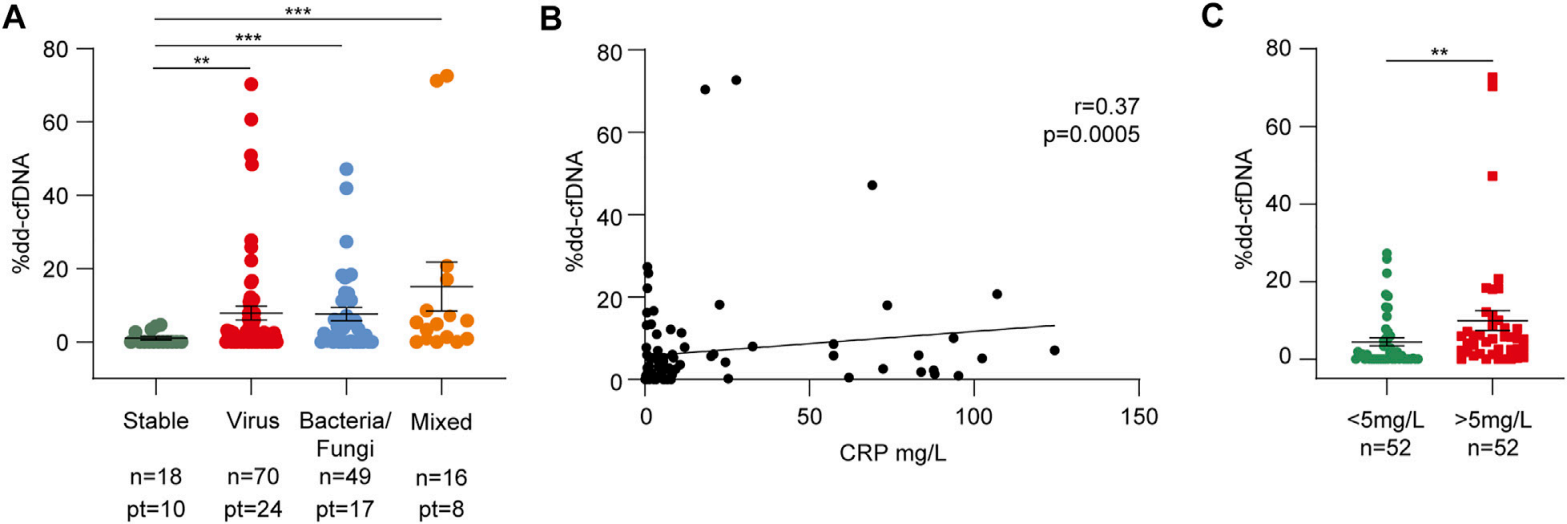
Respiratory tract infections cause dd-cfDNA release in the recipient bloodstream. **(A)** dd-cfDNA quantification related to infections divided into virus, bacteria/fungi, and mixed groups. The number of samples and patients (pt) from whom the samples were collected are shown for each category. **(B)** Linear regression between dd-cfDNA percentage and relative C-reactive protein (CRP) level (*n* = 104). Correlations were calculated using the nonparametric Spearman’s test. **(C)** Differences between %dd-cfDNA in the low (<5 mg/L) and high (>5 mg/L) CRP samples. The results in panels **(A)** and **(C)** are reported as percentages and shown as dot plots. Error bars represent SEM. *p*-values were obtained using the Mann-Whitney nonparametric test.

During follow-up, every patient experienced respiratory tract infections: bronchoalveolar lavage contained bacteria in 24 (80.0%), viruses in 23 (76.7%), and fungi in 11 (36.7%) cases, with specimens from eight patients (26.7%) showing mixed contamination (Table 1). Among the bacteria, the most frequent pathogens were *Pseudomonas aeruginosa* (12 specimens, 40.0%) and *Klebsiella pneumoniae* (5 specimens, 16.7%), while C*ytomegalovirus* (20 specimens, 66.7%) was the most common. Dd-cfDNA levels significantly increased during infectious episodes compared to stable conditions, with a slightly stronger increase observed during viral (7.70 ± 14.20%, *p* = 0.004) and mixed infections (13.7 ± 23.5%, *p* = 0.0007, Figures 3B, 4A; Supplementary Figure S2). Consistently, dd-cfDNA levels showed a positive association (*r* = 0.37, *p* = 0.0005) with C-reactive protein (CRP) blood levels, as determined by studying dd-cfDNA levels in 104 samples from 28 patients and collected close to CRP measurements during infection episodes (Figure 4B). One time point was excluded from the analysis because its CRP value was >300 mg/L, representing a potential bias in the statistical analysis. Considering 5 mg/L as a clinical cut-off value, the same samples were divided into low and high CRP groups. With this classification, samples collected from patients with CRP levels ≥5 mg/L showed significantly higher mean dd-cfDNA percentages (9.91 ± 16.4%) than low CRP samples (4.44 ± 7.13%, *p* = 0.004, Figure 4C).

### Donor-Derived Cell-Free DNA Percentages Correlate With Respiratory Function

**FIGURE 5:**
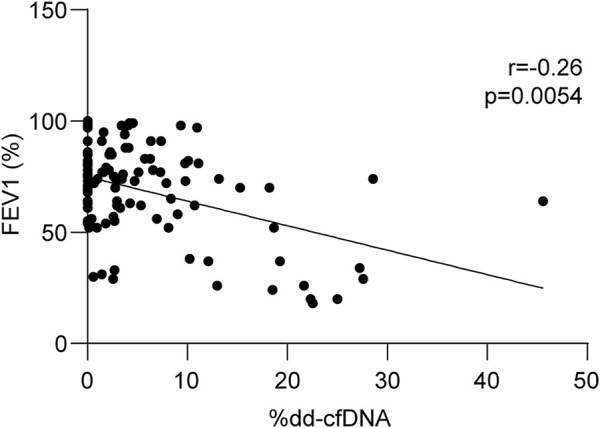
Dd-cfDNA is inversely related to respiratory function. Linear regression between forced expiratory volume in 1 s (FEV1) and related dd-cfDNA levels (*n* = 114). FEV1 was calculated considering recipient characteristics for normative equations. Correlations were obtained using the nonparametric Spearman test.

Lung transplant function was assessed using spirometry. FEV1 was quantified using recipient characteristics for the normative equation and considered a respiratory function measure. A total of 114 liquid biopsies were collected close to the spirometry tests, and dd-cfDNA quantification was correlated with relative FEV1 percentages. As shown in Figure 5, there was a statistically significant inverse relationship between the two variables (*r* = −0.26, *p* = 0.0054).

### Accuracy of the Test

**FIGURE 6:**
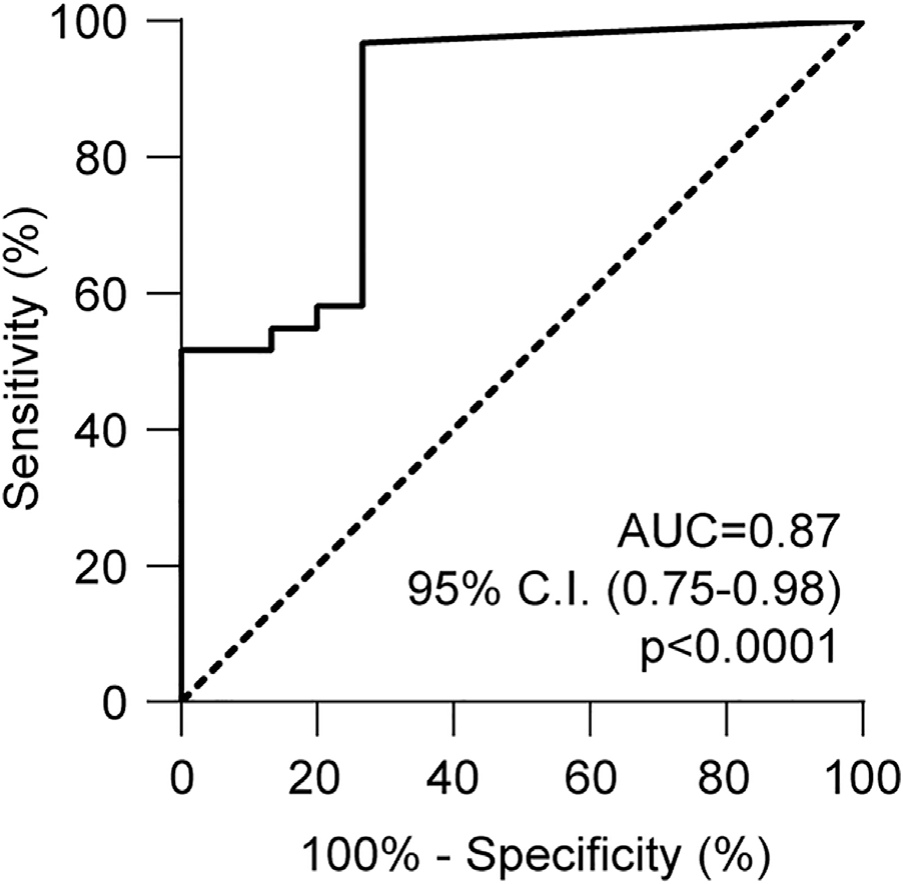
ROC analysis of *HLA-DRB1* droplet digital PCR assay. The ROC curve was obtained considering the dd-cfDNA values associated with rejection and no rejection. The curve was calculated using the Wilson-Braun method. Area under the curve (AUC) = 0.87 (95% C.I., 0.75–0.98).

Receiver operating characteristic (ROC) analysis was performed to assess the performance of this method. The area under the curve (AUC) was 0.87, (95% confidence interval: 0.75–0.98, *p* < 0.0001, Figure 6). With a cut-off value of 1.25%, dd-cfDNA had 80.7% sensitivity and 73.3% specificity for distinguishing AR from non-rejection. In particular, the test correctly identified 25 of the 31 biopsies classified as positive for rejection, and by excluding samples in which rejection occurred together with infection, dd-cfDNA quantification was above the cut-off value in 14 of 16 (87.5%) biopsies.

## Discussion

Long-term survival in lung-transplanted patients is limited by acute and chronic rejection, which represent the leading cause of graft failure and death, together with non-cytomegalovirus infections (6). Presently considered the gold standard tools for rejection monitoring, TBB and serum DSA evaluation show significant limitations in terms of sensitivity and specificity, thus limiting precise early diagnosis of graft damage and correct modulation of the immunosuppressive regimen (33-35). In this context, dd-cfDNA is emerging as a valuable adjunct and a reliable indicator of acute rejection after transplantation of several solid organs (13, 14, 16, 36). dd-cfDNA increases during rejection episodes based on the severity of the damage, whereas it remains low to undetectable in stable patients. In addition, dd-cfDNA can be easily purified from blood samples, causing minimal discomfort and stress to patients.

Donor DNA can be distinguished from the recipient’s exploitation of any type of genetic polymorphism spread across the genetic code. *HLA* genes are among the most polymorphic, and since this genetic diversity can be the basis of rejection, HLA locus is routinely typed before organ transplant. While this procedure is needed to exclude recipients who are already immunized against HLA alleles of the donor, in the case of lung transplantation, it has no impact on donor-recipient selection. It is therefore very infrequent that recipient and donor are matched: in our cohort of 328 lung transplants from 2000 to 2020, no transplants were fully HLA matched, while 29 (8.84%) were fully matched at the *HLA-DRB1* locus. These donor-recipient pairs were excluded from our assay, as there would be no genetic differences to monitor. However, it should be noted that if *HLA-DRB1* is matched, rejection occurs less frequently, and survival rates are higher (37-40)(Supplementary Figure S4). We previously applied a dd-cfDNA quantification method based on a probe panel targeting *HLA-DRB1* alleles and showed that it could identify rejection episodes in a cohort of heart-transplanted patients (23). The current technical improvement is that the same probe panel is now bound to two alternative fluorophores, FAM or HEX, and the differently labelled probes are loaded in the same reaction well, allowing the quantification of donor and recipient DNA percentages at the same time, thereby reducing costs by half while maintaining comparable sensitivity and specificity.

Compared to NGS, droplet digital PCR is more rapid, with results available 24 h after blood draw, feasible, and significantly cheaper. Using our optimized method, the cost of reagents for a single reaction is in the range of 80 euros, which is comparable to that of the Luminex assay for DSA monitoring (15, 23, 41).

The mean donor DNA percentages showed a clear correlation with the amount of donor tissue transplanted; bilateral transplant samples showed values approximately double those of single-lung transplants. Moreover, samples from patients who received more than one organ presented a significantly higher amount of dd-cfDNA, reflecting the higher number of donor cells inside the recipient. All samples collected in the first 2 weeks after transplantation demonstrated high levels of dd-cfDNA, consistent with ischemia-reperfusion injury and in line with previous data reported in the literature (5, 17, 33).

Donor DNA kinetics exhibited low percentages in samples collected from patients in stable conditions (Supplementary Figure S2), whereas values increased significantly in relation to ACR episodes, with moderate A2 rejection associated with a stronger release than A1 rejection. In addition, samples from patients with clinical signs of possible CLAD showed the highest dd-cfDNA levels. In particular, one of the CLAD patients developed early A2 rejection after 1-month post-transplant, and then suffered from relapsing pneumonia and chronic rejection treated with immunosuppressive boluses and photopheresis. Finally, the patient developed severe pulmonary insufficiency as a consequence of graft failure and died on post-transplant day 343. His dd-cfDNA levels increased early and did not decrease even after immunosuppressive treatment, consistent with severe rejection. Regarding the other two cases of CLAD, no specimens were collected after the additional immunosuppressive treatment, therefore no information could be obtained about their dd-cfDNA variations.

Morphological and immunohistochemical evaluations did not report evidence of AMR in any TBB collected during the follow-up period, even though seven recipients developed DSA. Although we observed a significant difference between DSA-positive samples and stable conditions, the reduced number of samples do not allow any speculation about the value of dd-cfDNA as a biomarker of AMR.

Remarkably, dd-cfDNA also increased in the presence of infection, in keeping with the notion that it is a marker of graft damage, independent of the cause (6). Therefore, for optimal clinical use, dd-cfDNA quantification should be performed together with a set of biomarkers of infection and radiological examination of the lung. The finding of increased dd-cfDNA in the absence of any sign of infection should prompt biopsy evaluation of the transplanted lung. Thus, dd-cfDNA could reduce the number of biopsies in a population of patients with a high suspicion of rejection.

In conclusion, we present an improved molecular method to monitor lung-transplant outcomes that allows rapid rejection identification through dd-cfDNA quantification at high costs. Larger clinical studies are needed to determine the best way to integrate this biomarker in the routine post-transplant management of lung transplant recipients to improve graft survival and patients’ quality of life.

## Data Availability

The original contributions presented in the study are included in the article/Supplementary Material, further inquiries can be directed to the corresponding author.

## Abbreviations

ACR, acute cellular rejection; AMR, antibody-mediated rejection; AR, acute rejection; AUC, area under the curve; BOS, bronchiolitis obliterans; CLAD, chronic lung allograft dysfunction; CRP, C-reactive protein; DCD, donation after circulatory death; dd-cfDNA, donor-derived cell-free DNA; DSA, donor-specific antibody; ECMO, extracorporeal membrane oxygenation; EVLP, *ex vivo* lung perfusion; FEV1, forced expiratory volume in 1 s; HLA, human leukocyte antigen; ICU, intensive care unit; ISHLT, International Society of Heart and Lung Transplantation; MFI, mean fluorescence intensity; n, number of samples; NGS, next-generation sequencing; PCR, polymerase chain reaction; PGD, primary graft dysfunction; ROC, receiver operating characteristics; SD, standard deviation; SNPs, single nucleotide polymorphisms; TBB, transbronchial lung biopsy.
