# Supplementary material for: Validation of a Simple, Rapid, and Cost-Effective Method for Acute Rejection Monitoring in Lung Transplant Recipients
Source: Transpl Int. 2022 Jun 9;35:10546. doi: 10.3389/ti.2022.10546 (PMC9221674; doi:10.3389/ti.2022.10546)
Supplement: Supplementary file 1 [file DataSheet1.docx]

**Supplementary material**

Table of content

[Supplementary figures 2](#_Toc96681990)

[Figure S1. Validation of HLA-DRB1 HEX probe panel. 2](#_Toc96681991)

[Figure S2. dd-cfDNA profile in 8 representative patients. 3](#_Toc96681992)

[Figure S3. Histopathological grades of acute rejection in our series. 4](#_Toc96681993)

[Figure S4. Lung transplant survival in 20 years of our experience. 5](#_Toc96681994)

[Supplementary tables 6](#_Toc96681995)

[Table S1. HLA Expert Design panel. 6](#_Toc96681996)

Supplementary figures


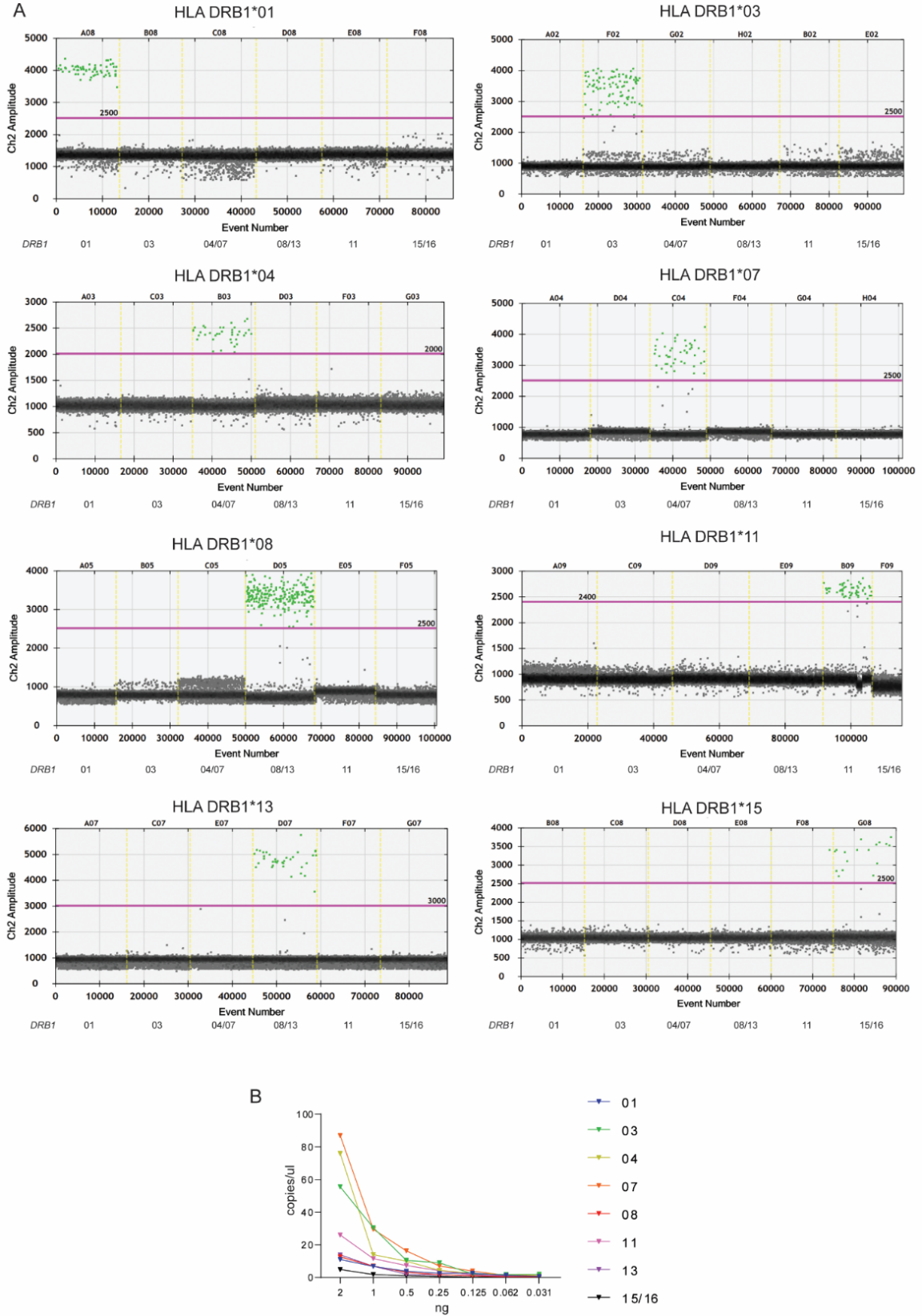


Figure S1. Validation of HLA-DRB1 HEX probe panel. A: all probes were tested for specificity by amplifying cfDNA carrying different combinations of HLA alleles. cfDNA typing loaded in each well is indicated below graphs. B: probe sensitivity was assessed by loading serial dilutions of cfDNA starting from 2ng up to 0.031ng.


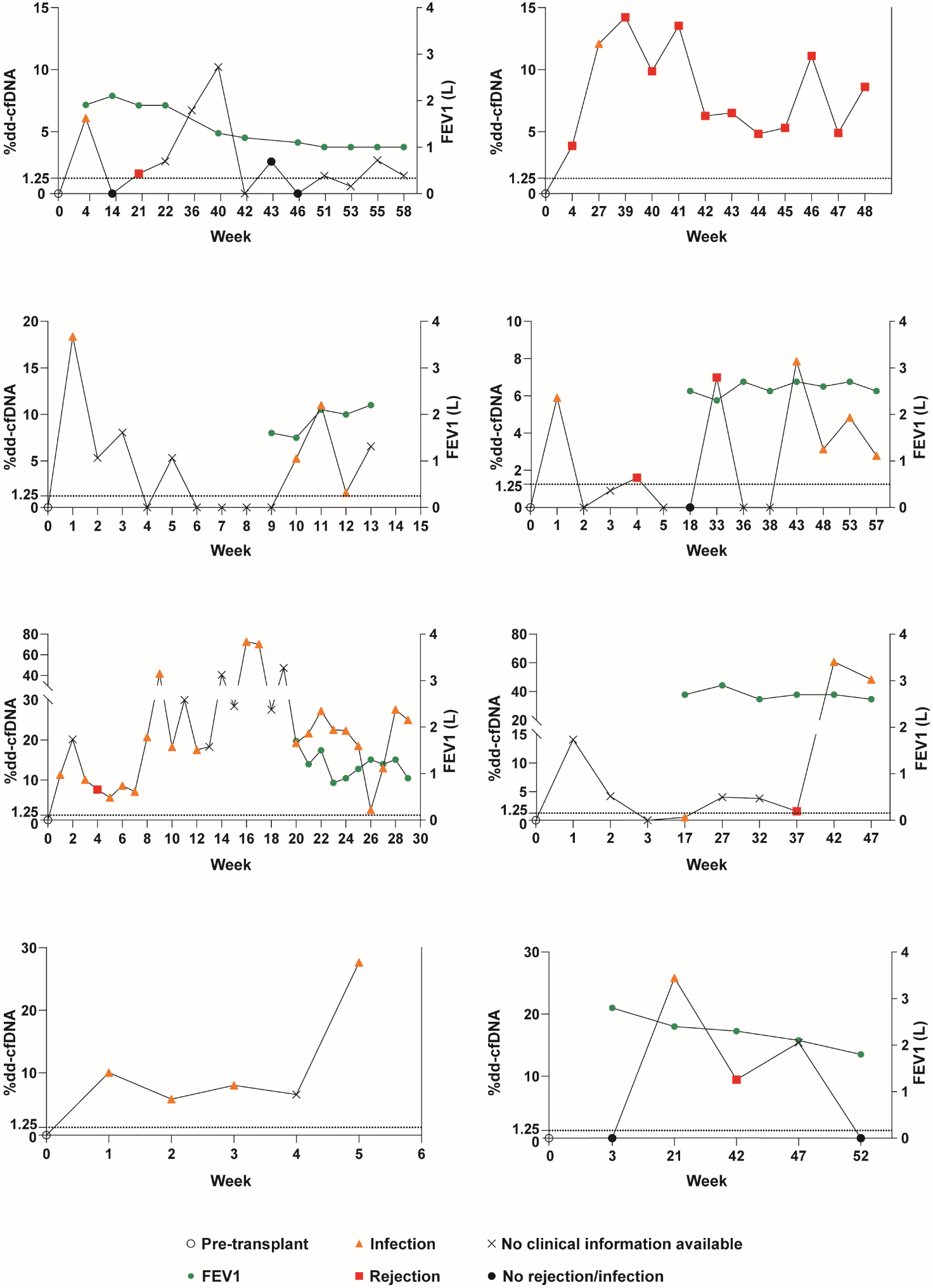


Figure S2. dd-**cfDNA profile in 8 representative patients.** dd-cfDNA levels during follow-up are shown. The presence of rejection (red square) and infection (orange triangle) is indicated. The positivity cut-off is set at 1.25% as resulted from ROC analysis. Available FEV1 measurements are reported as green dots.

**
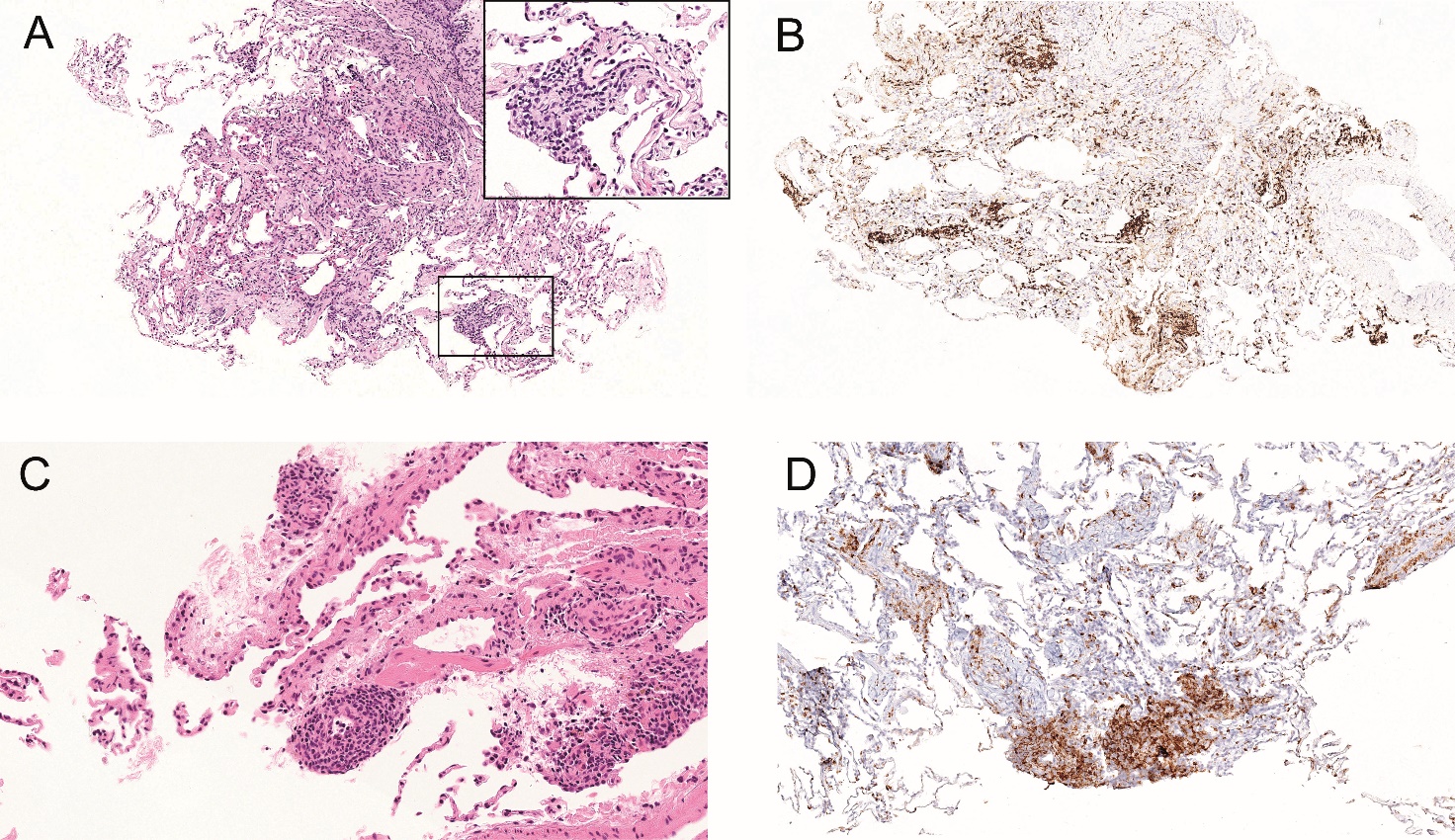
**

Figure S3. Histopathological grades of acute rejection in our series.

Grade A1 (panel A, hematoxylin and eosin staining, 100x original magnification) is characterized by a thin mononuclear inflammation focally surrounding small veins (focused on inset; 200x original magnification). T cells could be highlighted by CD3 immunohistochemical staining (panel B; 100x original magnification) that also showed the scattered involvement of venules within the lung parenchyma. Increased inflammatory cells, presence of eosinophils, and focal mild endothelitis characterized mild rejection (grade A2, panel C, hematoxylin and eosin staining, 200x original magnification); although there are more layers of lymphocytes organized around vessels, the surrounding alveolar interstitium is not involved, as highlighted by the CD3 immunohistochemical staining (panel D; 200x original magnification).

Figure S4. Lung transplant survival in 20 years of our experience.

3-year graft survival in *HLA-DRB1* matched (63%, n=154) and not matched (51%, n=159) donor-recipient pairs (p value=0.04).

# Supplementary tables

| **Probe ID** | **Target allele** | **Fluorophore** | **Amplicon length** |
| --- | --- | --- | --- |
| dHsaEXD29156242 | *HLA-DRB1*01* | FAM | 66 |
| dHsaEXD93426015 | *HLA-DRB1*03* | FAM | 70 |
| dHsaEXD67695788 | *HLA-DRB1*04* | FAM | 61 |
| dHsaEXD41965561 | *HLA-DRB1*07* | FAM | 66 |
| dHsaEXD16235334 | *HLA-DRB1*08* | FAM | 70 |
| dHsaEXD80505107 | *HLA-DRB1*11* | FAM | 98 |
| dHsaEXD54774880 | *HLA-DRB1*13* | FAM | 61 |
| dHsaEXD29044653 | *HLA-DRB1*15/16* | FAM | 67 |
| dHsaEXD29156242 | *HLA-DRB1*01* | HEX | 66 |
| dHsaEXD93426015 | *HLA-DRB1*03* | HEX | 70 |
| dHsaEXD67695788 | *HLA-DRB1*04* | HEX | 61 |
| dHsaEXD41965561 | *HLA-DRB1*07* | HEX | 66 |
| dHsaEXD16235334 | *HLA-DRB1*08* | HEX | 70 |
| dHsaEXD80505107 | *HLA-DRB1*11* | HEX | 98 |
| dHsaEXD54774880 | *HLA-DRB1*13* | HEX | 61 |
| dHsaEXD29044653 | *HLA-DRB1*15/16* | HEX | 67 |

# Table S1. HLA Expert Design panel.

List of the *HLA-DRB1* probes used in droplet digital PCR assays. Target allele, fluorophore and amplicon length are indicated for each probe.
